# Investigating AKT activation and autophagy in immunoproteasome-deficient retinal cells

**DOI:** 10.1371/journal.pone.0231212

**Published:** 2020-04-10

**Authors:** Md. Razaul Karim, Cody R. Fisher, Rebecca J. Kapphahn, Jorge R. Polanco, Deborah A. Ferrington

**Affiliations:** 1 Department of Ophthalmology and Visual Neurosciences, University of Minnesota, Minneapolis, Minnesota, United States of America; 2 Graduate Program in Biochemistry, Molecular Biology and Biophysics, University of Minnesota, Minneapolis, Minnesota, United States of America; University of Florida, UNITED STATES

## Abstract

Two major proteolytic systems, the proteasome and the autophagy pathway, are key components of the proteostasis network. The immunoproteasome, a proteasome subtype, and autophagy are upregulated under stress conditions, forming a coordinated unit designed to minimize the effect of cell stress. We investigated how genetic ablation of the LMP2 immunoproteasome subunit affects autophagy in retinal pigment epithelium (RPE) from WT and LMP2 knockout mice. We monitored autophagy regulation by measuring LC3, phosphorylation of AKT (S473), and phosphorylation of S6, a downstream readout of AKT (mTOR) pathway activation. We also evaluated transcription factor EB (TFEB) nuclear translocation, a transcription factor that controls expression of autophagy and lysosome genes. WT and LMP2 KO cells were monitored after treatment with EBSS to stimulate autophagy, insulin to stimulate AKT, or an AKT inhibitor (trehalose or MK-2206). Under basal conditions, we observed hyper-phosphorylation of AKT and S6, as well as lower nuclear-TFEB content in LMP2 KO RPE compared with WT. AKT inhibitors MK-2206 and trehalose significantly inhibited AKT phosphorylation and stimulated nuclear translocation of TFEB. Starvation and AKT inhibition upregulated autophagy, albeit to a lesser extent in LMP2 KO RPE. These data support the idea that AKT hyper-activation is an underlying cause of defective autophagy regulation in LMP2 KO RPE, revealing a unique link between two proteolytic systems and a previously unknown function in autophagy regulation by the immunoproteasome.

## Introduction

Maintenance of protein homeostasis, coined proteostasis, is essential for normal cellular function and in recovery from environmental insults or other stressors [1]. A key component involves the degradation of misfolded or damaged proteins that are produced during cell stress. The two distinct catabolic systems of proteostasis are the autophagy pathway and the proteasome, both of which are activated after cellular stress. The autophagy pathway consists of multiple steps starting with the formation of a double-membrane autophagosome that surrounds targets destined for degradation and ending with fusion with the lysosome, where sequestered molecules are degraded by acid hydrolases [2]. This pathway is responsible for degrading long-lived proteins, protein aggregates, and organelles [3]. Autophagy is stimulated by nutrient deprivation and multiple cellular stressors, including oxidative and ER stress, damage to DNA and organelles, accumulation of protein aggregates, and the presence of intracellular pathogens [4]. The proteasome is a multi-subunit complex that is responsible for degrading damaged and short-lived proteins as well as regulating critical cell processes, such as the cell cycle, signal transduction, and gene expression [1]. A proteasome subtype, known as the immunoproteasome, is upregulated under conditions of cell stress [5]. The immunoproteasome is defined by the inducible catalytic subunits, LMP2 (β1i), MECL-1 (β2i), and LMP7 (β5i), which are distinct from the catalytic subunits (β1, β2, β5) found in the 20S core of the standard proteasome [5].

Disruptions to autophagy or the immunoproteasome can have particularly devastating consequences in post-mitotic cells, such as the retinal pigment epithelium (RPE), a monolayer of cells that forms the blood-retina barrier. The RPE serves many physiological roles to maintain homeostasis of the retina, and is the primary site of defect in age-related macular degeneration (AMD), the number one cause of blindness in the elderly [1,6]. Studies of RPE from AMD donors have shown decreased autophagy flux [7] and in the retinas of AMD donors increased immunoproteasome content and activity has been observed [8]. Furthermore, genetic ablation of immunoproteasome subunits in mice hinders the ability of RPE to resist stress and disrupts cellular signaling [9,10,11].

One of the upstream regulators of autophagy is RAC-alpha serine/threonine-protein kinase (AKT), a protein kinase that controls a wide range of physiological responses, including metabolism, cell proliferation, and survival [12]. AKT regulates autophagy through mTOR and also through an mTOR-independent mechanism by controlling transcription factor EB (TFEB) nuclear translocation [13]. TFEB is the master transcription factor for the Coordinated Lysosomal Expression and Regulation (CLEAR) gene network, which encodes for autophagy and lysosomal proteins. Relevant to this study, knockout of the LMP2 immunoproteasome subunit in RPE increased PTEN content and decreased AKT phosphorylation relative to WT RPE following IGF treatment [11]. This result provided the first indication that a disruption of the immunoproteasome may alter AKT signaling, potentially affecting autophagy.

Evidence supporting the idea of coordinate interaction between the proteasome and autophagy includes multiple studies showing that disruption or inhibition of one catabolic system results in the compensatory activation of the other [14,15]. In this study, we investigated the regulation of the immunoproteasome and the autophagy pathway by comparing RPE from WT and LMP2 deficient mice. The LMP2 KO was selected based on previous studies showing the LMP2 KO elicited the greatest change in the stress response or signaling pathways compared with knockout of other immunoproteasome subunits [11,16,17]. Herein, we report over-activation of AKT signaling and an altered response to treatments that regulate autophagy. Additionally, we report that increased AKT signaling in LMP2 KO cells alters the content of nuclear TFEB, a potential mechanism for regulation of autophagy in RPE cells. These results show a unique link between two proteolytic systems that mutually contribute to maintenance of cellular proteostasis under stress.

## Material and methods

### Reagents

Dulbecco’s Modified Eagle’s medium (DMEM) and Earle’s balanced salt solution (EBSS) from Gibco (11965–092 and 24010–043). Chloroquine diphosphate (CQ), RIPA buffer, insulin, and trehalose dihydrate from Sigma-Aldrich (C6628, R0278, I9278, and T9531). MK-2206 2HCl (Selleckchem.com, S1078).

### Cell culture

Cell culture was done as reported previously [11]. In brief, RPE cells that were isolated from WT or LMP2 immunoproteasome KO mice were immortalized as described previously [18]. Cells were cultured in full (nutrient-enriched) media that contains DMEM with 5% or 10% heat-inactivated fetal bovine serum (Atlanta Biologicals, S11150H) for RPE and HEK-293, respectively with 1% penicillin-streptomycin (Gibco, 15070–063), and 1% sodium pyruvate (Gibco, 11360–070) under humidified conditions with 5% CO_2_ at 37°C. For autophagy flux studies 30μM CQ was added where indicated for 4 hours. Cells were treated with EBSS for 4 hours, 100nM insulin for 30 minutes, Trehalose (0, 25, 50, or 100mM) for 48 h, or 1μM MK 2206 for 24 hours.

### Western blotting

Cell were harvested using RIPA lysis buffer (Sigma Aldrich, R 0278) with protease inhibitor cocktail (Roche Molecular Biochemicals, 04693159001) and phosphatase inhibitor cocktails (Sigma-Aldrich, P5726 and P0044). Lysates were centrifuged at 12,000×g for 10 min. A total of 10μg of protein was separated by SDS polyacrylamide gel electrophoresis then transferred to nitrocellulose membrane and incubated overnight at 4°C with primary antibodies (S1 Table). The membrane was subsequently incubated with horseradish peroxidase-conjugated secondary antibody. Protein content was determined from the chemiluminescence signal and captured using a ChemiDoc XRS (Bio-Rad) and quantified using ImageJ (NIH) and normalized to the Ponceau stained blots as the loading control.

### Immunofluorescence and confocal microscopy

For immunostaining, cells were fixed then blocked with 3% normal goat serum (Sigma-Aldrich, G9023) after being permeabilized with 0.2% TBS-T for 15 min. Cells were incubated overnight at 4°C with primary antibodies followed by fluorescence-conjugated secondary antibodies (1:500; Alexa Fluor 647) for 2 h at room temperature in the dark and mounted. Cells were imaged using a confocal microscope (Olympus Fluoview FV-1000).

### eGFP-LC3 puncta counting

Both WT and LMP2 KO cells expressing LC3-GFP were fixed and imaged at 10x magnification. Images were then analyzed by an unbiased researcher to count the number of GFP puncta in each cell. An average of six images were taken per well, with an average of 16 cells per image over four replicate experiments.

### TFEB translocation image analysis

An average of 1484 (±24) cells were counted for each category. Image analysis and cell counting was completed using Image J. Total cell count was measured using the DAPI staining. Nuclear (Nuc)-TFEB positive cells were those that staining only in nucleus and fluorescence intensity was between 100–255 thresholds with a size larger than 30 μm^2^. To ensure cells were not counted twice, nuclear and cytosolic (N/C) dual distributed cells were those with fluorescence intensity from 65–255, minus the nuclear positive cell count. To further ensure cells were not counted repeatedly cytosolic (Cyto) cells were those which remained uncounted in both previous categories. Macros used for cell counting are available upon request.

### Nuclear/Cytosolic fractionation

Subcellular fractionation was carried out as described [19] with a slight modification. After treatments, cells were washed with ice cold PBS, lysed in 0.5% Triton X-100 lysis buffer (50 mM Tris-HCl, 0.5% triton, 137.5 mM NaCl, 10% glycerol, 5 mM EDTA) plus protease and phosphatase inhibitors cocktail and kept on ice and centrifuged at 13,000 × g for 8 min. The pellet containing the nuclear fraction was lysed by mixing with 0.5% Triton X-100 lysis buffer and 0.5% SDS. The supernatant comprises the cytosolic fraction. Until analysis, separated fractions were stored at -80°C after boiling for 10 min.

### Statistical analysis

To test for statistical significance between treatment groups, data was log transformed and analyzed by one-way or two-way analysis of variance (ANOVA) followed by a Multiple Comparison Post-hoc test (Tukey’s/Dunnett’s/Sidak’s), Fisher’s LSD with Holm’s correction, or Student’s *t* test using GraphPad PRISM Software (7.0). All the data are expressed as means ± Standard Error of the Mean (SEM) relative to WT controls. Probability (p) values less than 0.05 were considered significantly different. TFEB translocation count data was entered into R. Multinomial regression was used to generate p-values (S2 Table).

## Results

### Impaired autophagy and altered AKT signaling in LMP2 KO RPE

**Fig 1:**
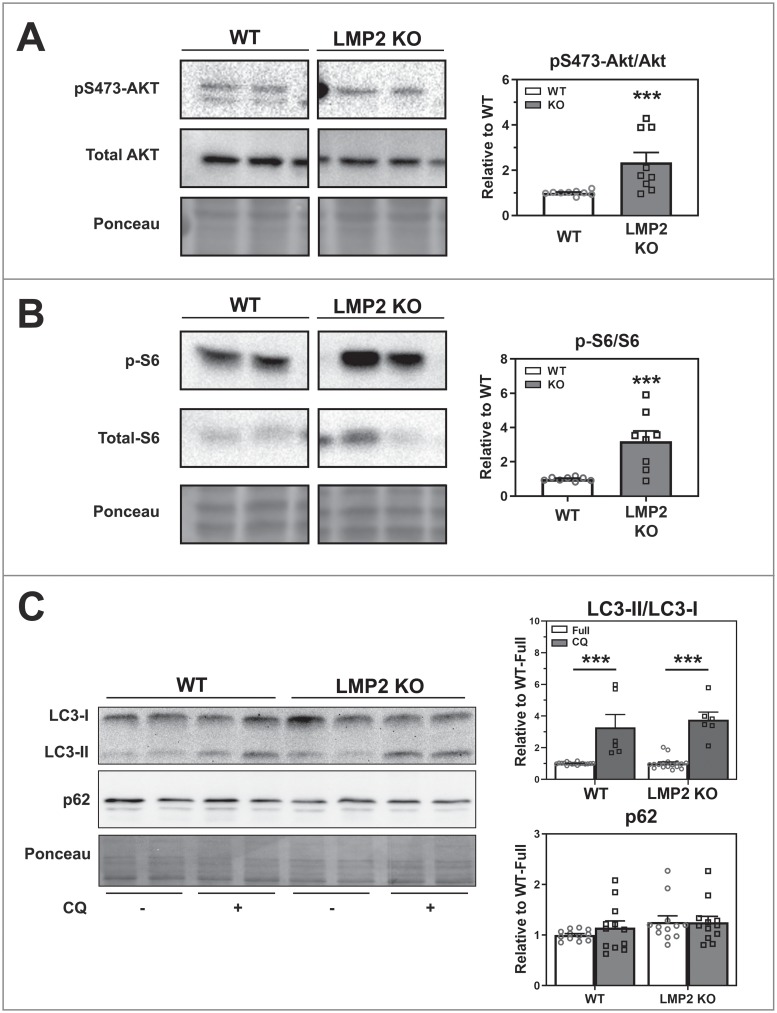
Comparing basal levels of AKT signaling and autophagy in mouse RPE cell cultures. **(A)** S473 phosphorylation state of AKT (n = 9) and **(B)** phosphorylation of S6 (n = 8) was assessed using Western Blot from mouse RPE under basal (full media) conditions. **(C)** LC3-II/I ratio and p62 content was analyzed from mouse RPE cultures under full media conditions with or without chloroquine (CQ) treatment. A representative blot (left) is shown for WT (n = 6–16) and LMP2 KO (n = 12) RPE cells. Bar graphs (right) summarize results from densitometry quantification of immune reactions normalized to WT-Full. All data is presented as mean ± SEM. Ponceau-stained blots served as the loading control. Significance assessed by Student’s t-test (**A, B**) and 2-Way ANOVA with uncorrected Fisher’s LSD followed by Holm’s correction on comparisons of interest (**C**). * denotes p<0.05, ** denotes p<0.01, and *** denotes p<0.001.

Previous work from our lab reported changes to phosphatidylinositol 3,4,5-trisphosphate 3-phosophatase and dual-specificity protein phosphatase (PTEN), an antagonist of the AKT pathway, and AKT phosphorylation in LMP2 KO RPE following treatment with IGF [11]. Since the AKT pathway regulates autophagy, we investigated AKT content and phosphorylation state in WT and LMP2 KO RPE (S1 Fig) under basal (full media) conditions. These cultured RPE, shown previously to express RPE65 [16], contain Bestrophin (BEST) and Cellular retinaldehyde–binding protein (CRALBP), two additional RPE-specific proteins (S1 Fig). Using immunoblotting, we evaluated the content of LC3, a protein involved in autophagosome formation. Monitoring the ratio of lipidated (LC3-II) to non-lipidated (LC3-I) forms provides a semi-quantitative assessment of autophagy [20]. Our results show that AKT was significantly hyper-phosphorylated (p = 0.002) in LMP2 KO RPE (Fig 1A). To monitor downstream signaling of AKT through mTOR, we checked phosphorylation of ribosomal S6, a protein responsible for activating protein synthesis. In the LMP2 KO cells, phospho-S6 was significantly (p<0.001) increased (Fig 1B). However, overactive AKT, which should have inhibited autophagy, had no effect on autophagy in LMP2 KO cells as seen from the LC3-II/I ratio and p62 that were comparable to WT levels (Fig 1C). CQ treatment, which allows LC3-II to accumulate because it impairs fusion of phagosomes with lysosomes, significantly (p<0.001) increased LC3-II/I ration in WT and LMP2 KO RPE. Taken together, these results indicate that AKT signaling is activated under basal conditions in LMP2 KO cells. However, this activation does not inhibit autophagy suggesting a potential defect in regulating autophagy.

### EBSS stimulation of autophagy

**Fig 2:**
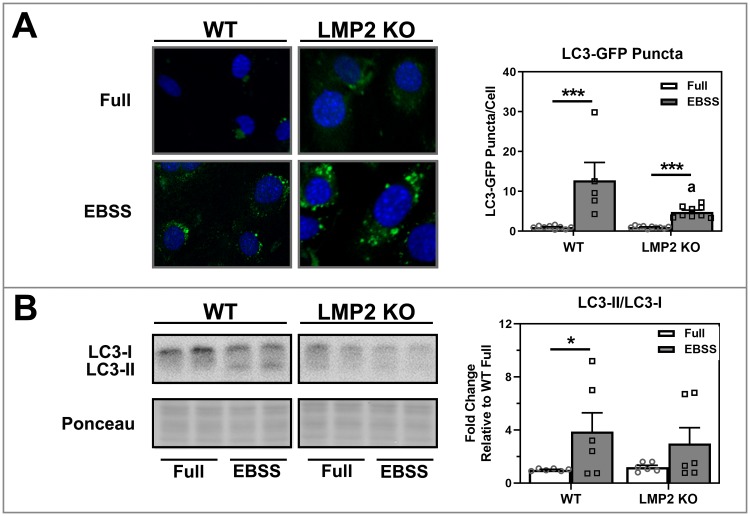
Assessment of autophagy following EBSS in RPE. **(A)** Confocal microscopy image of EGFP conjugated LC3 in RPE cells. pEGFP-LC3 stably transfected RPE cells were incubated with full or starvation medium (EBSS) for 4 hours. Puncta were counted and are presented as LC3-GFP puncta/cell (n = 5–10) **(B)** WT and LMP2 KO mRPE cells were analyzed for LC3 protein using Western immunoblotting (left). Bar graphs (right) show the ratio of LC3-II to LC3-I derived from densitometry analysis of immunoblots (n = 6). Data are means ± SEM. Data were analyzed by 2-Way ANOVA with uncorrected Fisher’s LSD followed by Holm’s correction on comparisons of interest. * denotes p<0.05, ** denotes p<0.01, and *** denotes p<0.001. a denotes p<0.05 from respective WT groups.

To further investigate potential defects in autophagy regulation in LMP2 KO cells, we tested autophagy following starvation with EBSS, a common method of autophagy induction that by-passes AKT. The presence of LC3-EGFP puncta was monitored to visually confirm that the treatment upregulate autophagy. RPE cells from WT and LMP2 KO mice were transfected with EGFP-LC3 plasmid before incubation with either complete nutrient-enriched (full) media or EBSS (Fig 2A). Under starvation conditions LC3-puncta accumulated in both WT and LMP2 KO cells. However, the extent of upregulation was substantially different; LC3-GFP puncta were increased 12-fold in WT (p<0.001) but only 5-fold in KO (p<0.001).

To further validate these initial results of the effect of starvation, Western blot was used to quantify the LC3-II/I ratio as a measure of autophagy (Fig 2B). We found EBSS significantly increased the LC3-II/I ratio by 4-fold in WT (p = 0.04), but in LMP2 KO RPE, the LC3-II/I ratio was elevated only 3-fold over full media conditions (p = 0.25). These results showing reduced autophagy induction suggest dysfunction of this process under starvation-induced stress in LMP2 KO cells.

### Insulin stimulation of AKT

**Fig 3:**
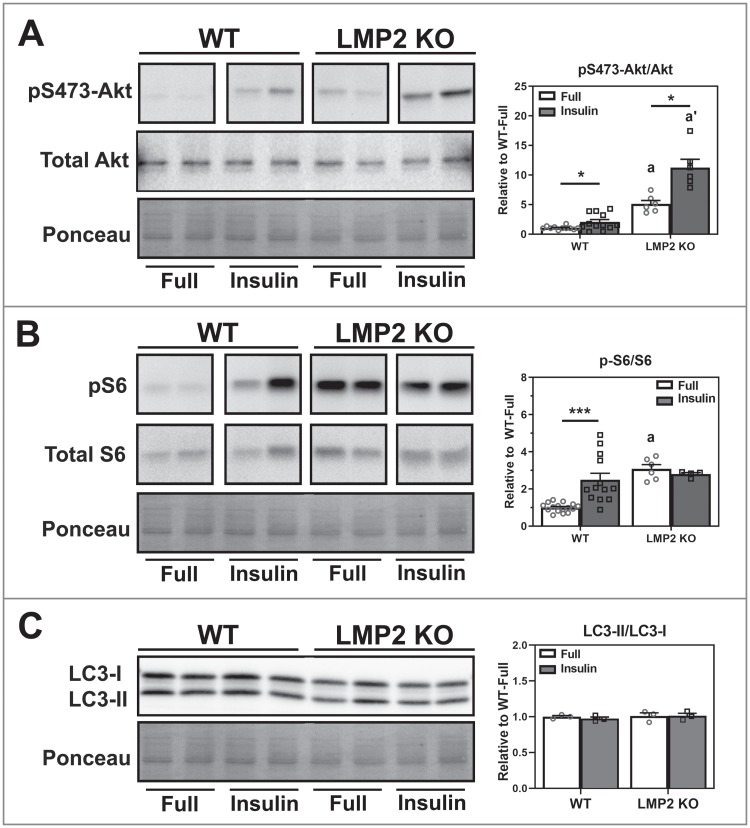
Insulin stimulation of AKT in RPE cells from WT and LMP2 KO mice. Total lysates were collected from mouse RPE cultured in either basal (full media) conditions or after treatment with 100nM insulin for 30 minutes. Representative blots (left) and densitometry quantification of immune reactions (right) are shown. **(A)** Phosphorylation state of AKT was assessed via Western blot (n = 7–12). **(B)** S6 phosphorylation was measured following insulin treatment in both cells to assess activation of the AKT pathway (n = 4–16). **(C)** LC3-II/I ratios were quantified for both WT and LMP2 KO cells following insulin treatment (n = 3). All data are mean ± SEM and normalized to WT-Full. Ponceau-stained blots served as the loading control. Significance assessed by 2-Way ANOVA with uncorrected Fisher’s LSD post-hoc test followed by Holm’s correction on comparisons of interest. * denotes p<0.05 and *** denotes p<0.001. a and a’ denote p<0.05 from respective WT groups.

Having established a defect in autophagy regulation, we next used insulin to stimulate AKT to further define the site of defect. After insulin treatment, we observed a 2-fold increase in AKT phospho-S473 in both WT (p = 0.046) and LMP2 KO (p = 0.013) cells (Fig 3A). As expected, ribosomal S6 phosphorylation was significantly increased following insulin treatment in WT (p<0.001) (Fig 3B). However, the phosphorylation state was unchanged in LMP2 KO RPE (Fig 3B). A potential explanation for the apparent lack of response could be that under basal conditions, phospho-S6 was already elevated in LMP2 KO cells to a level comparable to WT after insulin treatment. Therefore, it is possible that insulin could not further activate S6. While AKT activation should inhibit autophagy, no change was observed in LC3-II/I ratio after insulin treatment in both WT and LMP2 KO cells compared to basal (full media) conditions (Fig 3C). This is likely because minimal autophagy would be ongoing under full media conditions and any decrease may not be detected with this assay. These results suggest that AKT was equally responsive to insulin in both cell types, and a lack of downstream response was due to the initial over-activation of AKT.

### AKT inhibition increases autophagy

**Fig 4:**
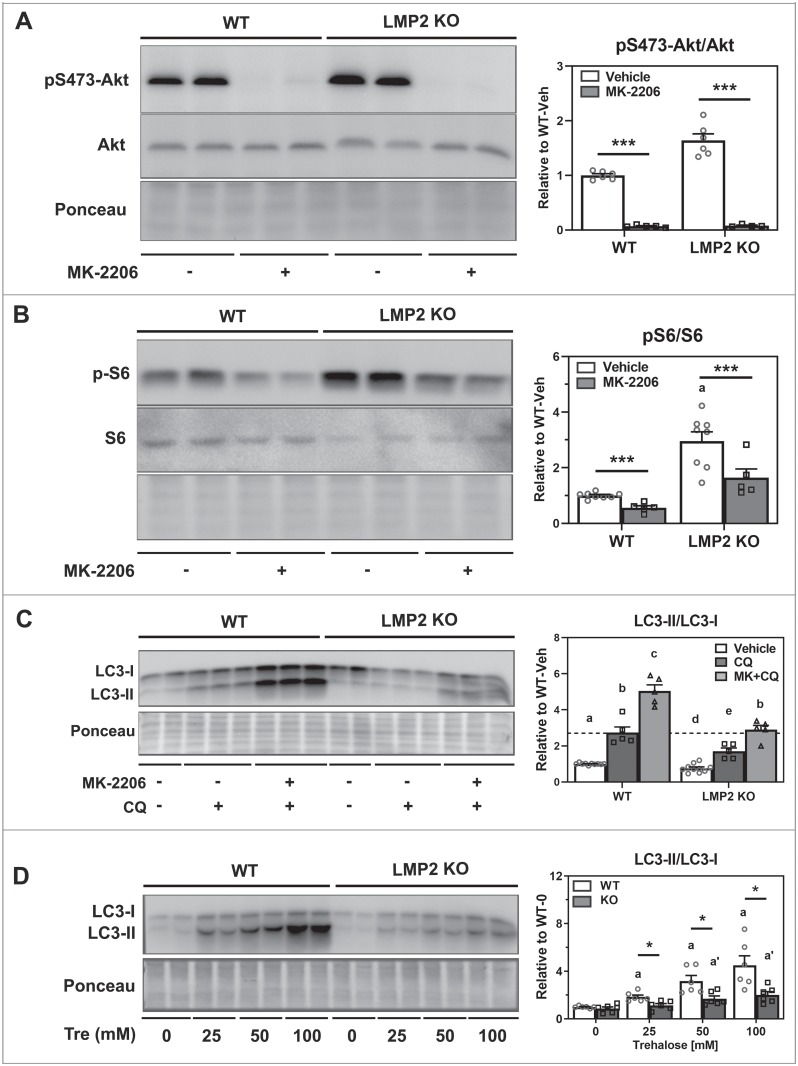
Inhibition of AKT phosphorylation and its impact on autophagy. Total lysates were collected from WT and LMP2 KO RPE cells treated with either 1μM MK-2206 (A, B, C) or trehalose at varying doses (D). Representative immune reactions (left) and bar graph summarizing densitometry (right) are presented. **(A)** Phosphorylation of the S473 site of AKT was assessed via Western blotting (n = 4–6). **(B)** Phosphorylation S6 was assessed following treatment with MK-2206 (n = 5–8). **(C)** Cells were maintained with full medium (n = 5–11) with 1μM MK-2206 for 24 h in the presence (n = 5) or absence (n = 5) of 30 μM CQ for 4 h. ^a-e^Different letters indicate significant difference at p<0.05 from uncorrected Fisher’s LSD post-hoc test followed by Holm’s correction on comparisons of interest. **(D)** RPE from WT and LMP2 KO mice were cultured with varying doses of trehalose (0-100mM) before total lysates were collected (n = 5 for each group). LC3-II/I ratios were quantified. All data are presented as mean ± SEM normalized to their WT controls. * denotes p<0.05 and *** denotes p<0.001. a and a’ denote p<0.05 from respective WT groups.

To follow up on our initial observation of increased AKT phosphorylation under basal (full media) conditions in LMP2 KO RPE (Fig 1C) we tested how AKT inhibition affects downstream processes. We used MK-2206, a potent kinase inhibitor of AKT, and trehalose, a disaccharide that enhances autophagy using an mTOR-independent mechanism [13,20,21]. MK-2206 had a significant inhibitory effect on AKT phosphorylation (p<0.001) (Fig 4A and S2A Fig). MK-2206 also caused a significant (WT: p = 0.002, KO: p = 0.002), but not complete, decrease in phosphorylation of S6 in both cell types (Fig 4B). Western blotting was used to quantify LC3 after incubating cells with CQ and MK-2206 to evaluate autophagy flux (Fig 4C). Overall, LC3-II/I ratios were lower in LMP2 KO cells. However, MK-2206 induced a significant 2-fold increase relative to their CQ controls, showing that both WT (p<0.001) and LMP2 KO (p<0.001) were responsive to inhibition of AKT. Of note, comparison of the LC3-II/I ratio in WT cells treated with CQ alone and LMP2 KO cells treated with CQ and MK-2206 were equivalent (Fig 4C, dashed line). The incomplete loss of S6 phosphorylation suggests mTOR was affected by MK-2206 treatment, potentially contributing to the changes in autophagy.

In RPE, trehalose partially inhibited AKT phosphorylation, but had no effect on S6 phosphorylation (S2A and S2B Fig), supporting the idea that it acts through an mTOR independent mechanism. Trehalose’ effect on autophagy was verified by the dose-dependent increase in LC3-II/I ratio in RPE from both strains of mice (Fig 4D). However, there was a significantly more robust response from WT compared with LMP2 KO cells at 25 (p = 0.009), 50 (p = 0.002), and 100μM trehalose (p<0.001).

### AKT inhibition regulates TFEB translocation

AKT can also control autophagy via nuclear translocation of TFEB, a transcription factor that regulates genes associated with lysosomal biogenesis and autophagy. A recent study in Hek-293 cells showed that both AKT inhibitors, MK-2206 and trehalose, activate TFEB nuclear translocation by attenuating AKT activity [13]. These previous results in HEK-293 cells were replicated using our experimental conditions (S3 Fig).

**Fig 5:**
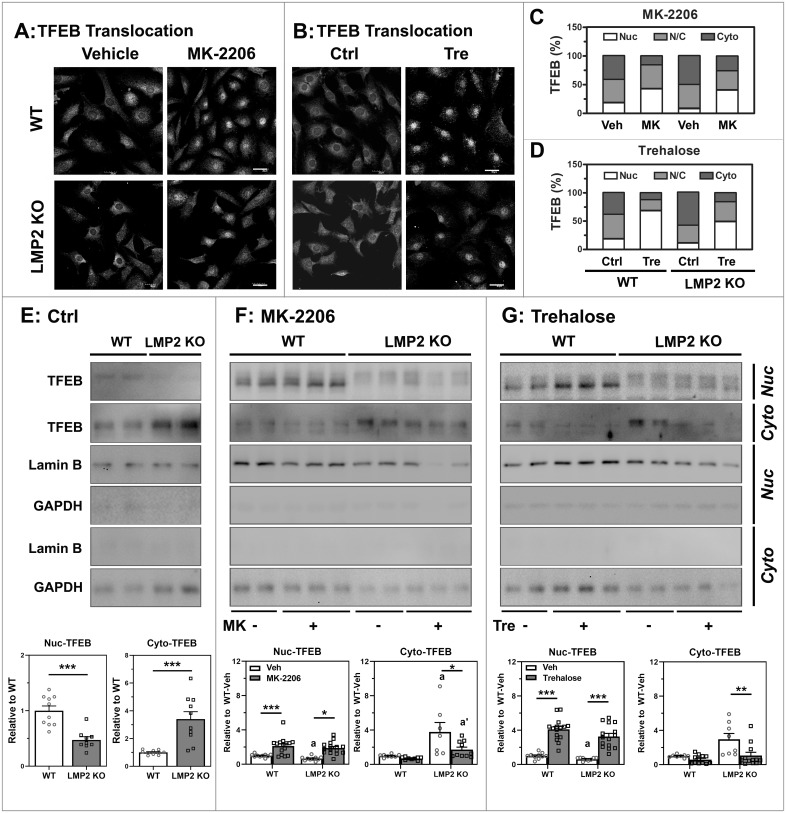
Inhibition of AKT phosphorylation and nuclear TFEB translocation. **(A, B)** TFEB translocation was monitored in cells immunostained with the TFEB antibody and captured by confocal microscope. Scale bar: 30 μm. **(C, D)** Cell counts for subcellular localization of TFEB were done from confocal microscope images. An average 1484 (±23) cells/sample were counted using automated Image J (Fiji). Nuclear (Nuc), cytosolic (Cyto) and dual nuclear-cytosolic (N/C) distribution of TFEB was determined as described in “Material and methods”. **(E, F, G)** Nuclear (Nuc) and cytosolic (Cyto) subcellular fractionation of RPE cells was used to monitor TFEB localization (n = 8–15). Data were analyzed via student’s t-test (E) or 2-Way ANOVA with uncorrected Fisher’s LSD post-hoc test followed by Holm’s correction for comparisons of interest (F, G). Data are presented as mean ± SEM normalized to respective WT controls. * denotes p<0.05, ** denotes p<0.01, and *** denotes p<0.001. a and a’ denote p<0.05 from respective WT controls.

To explore AKT’s effect on TFEB translocation, TFEB cellular localization was evaluated using confocal microscopy following treatment with either MK-2206 (Fig 5A) or trehalose (Fig 5B). Cells were categorized based on TFEB localization in the cytoplasm (Cyto) or nucleus (Nuc), or when TFEB signal was present throughout the cell (N/C). Data in Fig 5C and 5D summarizes the proportion of TFEB localized to specific cellular regions after MK-2206 or Trehalose treatment. Cell counts were statistically analyzed using multinomial logistic regression to identify significant differences in treatment response due to absence of the LMP2 subunit (S2 Table). Both MK-2206 and trehalose induced significant TFEB nuclear translocation. With MK-2206 treatment, WT and LMP2 KO cells showed a significantly different response in N/C cell count (Fig 5C, S2 Table). For trehelose treatment, WT and KO cells had a significantly different response in the proportion of cells for both Nuc and N/C counts (Fig 5D, S2 Table).

To validate image analysis of TFEB activation, we monitored TFEB translocation by Western blotting after subcellular isolation of nuclear and cytosolic fractions (Fig 5E–5G). Purity of the fractions was confirmed by the presence or absence of nuclear localized laminB and cytosolic glyceraldehyde-3-phosphate dehydrogenase (GAPDH). Under basal conditions, levels of Nuc-TFEB was significantly lower for LMP2 KO, consistent with lower total TFEB in whole cell lysates (S2C Fig). However, Cyto-TFEB was significantly increased in LMP2 KO compared to WT cells (p<0.001) (Fig 5E). As expected, hyper-activation of AKT prevented TFEB translocation in LMP2 KO cells, resulting in higher TFEB levels in the cytoplasm. Quantification of nuclear and cytoplasmic TFEB based on cell fractionation and Western blot is consistent with imaging results showing decreased nuclear TFEB and increased cytoplasmic TFEB in LMP2 KO RPE. Importantly, these imaging results are unaffected by total TFEB content.

Following treatment with MK-2206, significant translocation of Nuc-TFEB was observed for both WT and LMP2 KO cells. However, a significant (p = 0.027) reduction of Cyto-TFEB was found only in LMP2 KO cells (Fig 5F). Trehalose caused a significant upregulation of Nuc-TFEB translocation for both groups (Fig 5G). Cyto-TFEB levels were significantly different only in LMP2 KO cells (p = 0.007). The apparent lack of change in the Cyto-TFEB levels in WT cells for both treatments could be due to the insufficient sensitivity of Western blots in detecting small decreases in TFEB content when WT baseline levels were already low. Taken together, these data confirm that by inhibiting AKT, nuclear translocation of TFEB increases, thereby stimulating autophagy in both WT and LMP2-deficient cells (Fig 4C and 4D).

## Discussion

Herein, we investigated how genetic ablation of the LMP2 immunoproteasome subunit affects AKT phosphorylation state and subsequent downstream effects on autophagy. Our results provide evidence that the immunoproteasome and autophagy pathways communicate, and their site of regulation is AKT. Overall, we observed hyper-phosphorylation of AKT in LMP2 KO RPE under basal conditions (Fig 1), and subsequently lower nuclear TFEB content (Fig 5). Starvation of cells using EBSS revealed autophagy defects in LMP2 KO RPE (Fig 2). Decreasing AKT phosphorylation using MK-2206 or trehalose stimulated nuclear TFEB translocation in both cells types (Fig 5) and uncovered AKT’s role in autophagy regulation in LMP2 KO RPE (Fig 4). Taken together, our results support the notion that AKT hyper-activation is the major underlying cause of defective autophagy regulation in LMP2 KO RPE.

RPE cells, essential for retinal homeostasis, are a post-mitotic cell that ingests photoreceptor outer segments [22]. Outer segments are internalized and degraded through LC3-associated phagocytosis, a process that utilizes the autophagy machinery for their degredation [22]. Thus, there is an exceptional requirement for active proteolysis and autophagy to maintain RPE function. Several phenotypic changes in the RPE suggest defects in proteostasis with aging and AMD. For example, an age-related accumulation of lipofuscin, an aggregate of undigested lipids and proteins from outer segments, is observed in RPE of mice and humans [23,24]. With AMD, the autophagy capacity of the RPE becomes insufficient, as evidenced by reduced autophagy flux in primary RPE cultures from human donors [7]. These findings implicate defective proteostasis in the aged RPE and the pathology of AMD.

The proteasome, the other major proteolytic pathway, is also important in maintaining cellular function. A subtype of the proteasome, the immunoproteasome, is abundant in immune cells and has an important role in antigen processing and presentation [25]. The immunoproteasome is also present at low abundance in cells outside the immune system, including neurons, retina, RPE, skeletal muscle, and liver [1,9,16,26]. Notably, immunoproteasome content is significantly upregulated under conditions of stress, clearly linking this proteolytic complex to the stress response [9,26,27,28]. Studies in non-immune cells have shown the immunoproteasome functions to maintain protein homeostasis and cell viability under multiple stressors, including inflammatory cytokines and oxidative stress [1,28,29]. Specific to the retina and RPE, the immunoproteasome is upregulated by aging, retinal injury induced by optic nerve crush, oxidative stress, inflammatory cytokines, and with disease [5,8,9,10,11]

Mice with genetic ablation of immunoproteasome subunits have provided important details regarding the requirement for specific subunits in maintaining cell function. These knockout mice were originally developed by immunologists to investigate the role of specific immunoproteasome subunits in antigen presentation [30,31,32]. Investigations using these mice have discovered numerous putative functions for each subunit not only in immune cells, but also in non-immune cells where evidence suggests immunoproteasome’s function goes beyond antigen presentation [5,9,10,11,28,30,31,32]. One of the important observations in KO mice was the presence of an "intermediate" proteasome core containing a mixture of both the standard and immunoproteasome catalytic subunits. Of note, these intermediate core particles retain proteasome activity, as shown from activity measured in the spleen, where the immunoproteasome makes up the majority of the proteasome population, in mice lacking both LMP7 and MECL immunoproteasome subunits [9]. Subsequently, these intermediate proteasomes have been found in a range of WT cells and tissues, including liver, kidney, skeletal and cardiac muscle, thereby supporting the idea that different proteasome subtypes could have specific functions within the cell [33,34,35,36]. This idea is further supported by studies comparing the response of mice with different immunoproteasome subunits knocked out. For example, subunit-specific responses were observed in LMP2 versus LMP7/MECL KO cells following treatment with either TNFα or IGF-1, and in retinal survival of mice following optic nerve crush [11,16]. How the absence of different immunoproteasome subunits or the presence of specific intermediate 20S proteasome cores affects cell function remains one of the major questions in proteasome biology.

We used MK-2206 and trehalose to inhibit AKT. Both drugs were effective modulators of AKT signaling, ultimately increasing autophagy and nuclear TFEB translocation. TFEB is part of a family of transcription factors that include microphthalmia-associate transcription factor (MITF), transcription factor E3 (TFE3) and transcription factor EC (TFEC), which are all controlled by AKT phosphorylation [37]. Expression of TFEB, MITF, and TFE3 has been reported in RPE, but TFEC expression is restricted to cells of myeloid origin [37]. While we only tracked the translocation of TFEB, it is possible that MITF and TFE3 could have contributed to the observed changes in autophagy since all of these transcription factors can bind CLEAR network sites to induce autophagy and lysosome biogenesis [38]. However, we focused on TFEB because of its well-described mechanism involving AKT phosphorylation of TFEB at S467, which represses its nuclear transport. TFEB dephosphorylation allows for its nuclear translocation where it upregulates the CLEAR network and subsequently autophagy, independent of mTOR [13].

Abnormal AKT activation has been linked to multiple diseases, such as insulin resistance, metabolic disorders, and tumor growth [21]. Thus, investigators have explored using AKT modulators as therapeutics. A recent report described the clinical development of trehalose for targeting arterial aging, oculopharyngeal muscular dystrophy, spinocerebellar ataxia 3, and bipolar depression [13]. MK-2206 is currently in clinical trials as an anticancer agent [39]. Based on reports of decreased RPE autophagy with aging and AMD [7,24], these drugs may also be effective in reversing age-related and AMD autophagy deficits in the RPE.

While our study focused specifically on AKT, we did not explore all mechanisms linking the immunoproteasome to autophagy, which could be through either an indirect or a direct interaction of the two pathways. Potential indirect links include the changes in immunoproteasome activity in the LMP2 knockout that could allow for the accumulation of molecules that are toxic to or interfere with autophagy. As AKT is a hub of cellular signaling, its over activation in the LMP2 model may alter other signaling pathways that subsequently have a negative effect on autophagy. We also did not explore the mechanism behind AKT hyper-phosphorylation, which we believe drives the autophagy defect. AKT hyper-phosphorylation could be due to either over-activation of kinases that phosphorylate (phosphatidylinositol 4,5-bisphosphate 3-kinase (PI3K), 3-phosphoinositide dependent protein kinase 1 (PDPK1), or mTOR complex 2 (mTORC2)) or inhibition of phosphatases (protein phosphatase 2A (PP2A), PH domain leucine-rich repeat phosphatase (PHLPP) 1 and 2) that dephosphorylate these sites [40]. Of note, inhibition of the major phosphatases leads to AKT hyper-phosphorylation in many cancer cells [41]. Conversely, the changes to AKT and autophagy may be through direct interaction between proteins participating in each pathway. For example, PRAS40, a constituent of the protein complex mTOR, coordinates the synthesis of immunoproteasomes to protect cells against protein stress [42]. Investigation of the upstream kinases and phosphatases, and the downstream targets, such as mTOR, is required to understand the mechanistic link between these two proteostasis pathways.

In conclusion, our findings demonstrate that LMP2 subunit deficiency leads to an over activation of AKT and defects in autophagy regulation, suggesting that the LMP2 immunoproteasome subunit is a necessary molecule for maintaining normal autophagy. By inhibiting AKT phosphorylation, we partially rescued autophagy defects in LMP2 KO RPE through stimulation of TFEB nuclear translocation. Our results revealed a novel mechanism linking autophagy and the immunoproteasome via regulation by AKT.

## Supporting information

S1 Fig Immunoproteasome subunits and RPE specific proteins in cultured RPE. (EPS)

S2 Fig Akt’s response to trehalose and MK-2206 and total TFEB content in mRPE. (EPS)

S3 Fig TFEB translocation in HEK-293 cells. (EPS)

S1 Table (PDF)

S2 Table (PDF)

## Abbreviations

AKT: RAC-alpha serine/threonine-protein kinase
AMD: age-related macular degeneration
CQ: chloroquine
KO: knockout
LC3: microtubule-associated protein 1A/1B-light chain 3
LMP2: low-molecular-mass protein
mTOR: mammalian target of rapamycin complex 1
POS: photoreceptor outer segment
RPE: retinal pigment epithelium
S6: ribosomal protein S6
TFEB: transcription factor EB
