# Supplementary material for: Investigating AKT activation and autophagy in immunoproteasome-deficient retinal cells
Source: PLoS One. 2020 Apr 10;15(4):e0231212. doi: 10.1371/journal.pone.0231212 (PMC7147741; doi:10.1371/journal.pone.0231212)
Supplement: S1 Table — (PDF) [file pone.0231212.s004.pdf]

Table S1. Antibodies used for Western blotting and immunohistochemistry.

Antibody	Detect	Assay	Dilution	Catalog	Company
LC3B	M	WB	0.73611	2775	Cell Signaling
pS473-Akt	M	WB	0.73611	4060	Cell Signaling
Akt	M	WB	0.73611	4691	Cell Signaling
pS6	M	WB	0.73611	2211	Cell Signaling
S6	M	WB	0.38889	2317	Cell Signaling
TFEB	M	WB/IH	1:1000;1:400	10095-830	Proteintech
TFEB	H	WB	0.73611	4240	Cell Signaling
GAPDH	M	WB	0.38889	H86504M	Meridian Life Sc.
Lamin B	H/M	WB	0.73611	ab133741	Abcam
LMP2	M	WB	0.21528	sc-373996	Santa Cruz
MECL1	R	WB	0.73611	BML-PW8150	Enzo
LMP7	R	WB	0.73611	13635	Cell Signaling
α7	M	WB	0.73611	BML-PW8110	Enzo
