# Supplementary material for: Investigating AKT activation and autophagy in immunoproteasome-deficient retinal cells
Source: PLoS One. 2020 Apr 10;15(4):e0231212. doi: 10.1371/journal.pone.0231212 (PMC7147741; doi:10.1371/journal.pone.0231212)
Supplement: S2 Table — (PDF) [file pone.0231212.s005.pdf]

Table S1. Results of TFEB image analysis using multinomial logistic regression. Probability values were generated from cell counts in **Fig. 6C** (MK-2206) and **6D** (trehalose). Cells with only cytosolic (Cyto) TFEB immunofluorescence are used as the baseline to compare the dual nuclear-cytosolic (N/C) and nuclear (Nuc) TFEB distribution. p<0.05 values are considered as significant and are in bold.

Treatment Condition	Sub cellular Fraction	Statistical Analysis		
		Cell Type [a]	Treatment [b]	Cell Type : Treatment [c]
MK-2206	N/C	6.98E-02	1.78E-01	**4.24E-02**
	Nuc	5.79E-02	**<0.001**	3.91E-01
Trehalose	N/C	**3.09E-02**	**<0.001**	**5.01E-04**
	Nuc	**4.40E-14**	**<0.001**	**1.13E-02**

[a] Cell Type = WT and LMP2 KO.

[b] Treatment = MK-2206 or trehalose (untreated control Vs treatment).

[C] Cell Type : Treatment = Interaction between cell type and treatment.
